# Cross-sectional and longitudinal associations between psychotic and depressive symptoms in depressed adolescents

**DOI:** 10.1007/s00787-020-01704-3

**Published:** 2021-01-11

**Authors:** Fiona Kehinde, Aamena Valiji Bharmal, Ian M. Goodyer, Raphael Kelvin, Bernadka Dubicka, Nick Midgley, Peter Fonagy, Peter B. Jones, Paul Wilkinson, IMPACT Consortium

**Affiliations:** 1 School of Clinical Medicine, University of Cambridge, Cambridge, UK; 2 Department of Psychiatry, University of Cambridge, Douglas House, 18b Trumpington Road, Cambridge, CB2 8AH UK; 3 Cambridgeshire and Peterborough NHS Foundation Trust, Cambridge, UK; 4 Anna Freud National Centre for Children and Families, London, UK; 5 Research Department of Clinical, Educational and Health Psychology, University College London, London, UK; 6 University of Manchester, Manchester, UK; 7 Imperial GP VTS Scheme, London, UK; 8 Royal Free Hospital, London, UK

**Keywords:** Psychotic symptoms, Adolescence, Unipolar depression, Depression severity

## Abstract

Adults with major depressive disorder (MDD) with psychotic features (delusions and/or hallucinations) have more severe symptoms and a worse prognosis. Subclinical psychotic symptoms are more common in adolescents than adults. However, the effects of psychotic symptoms on outcome of depressive symptoms have not been well studied in adolescents. Depressed adolescents aged 11–17 with and without psychotic symptoms were compared on depression severity scores at baseline and at 28- or 42-week follow-up in two large UK cohorts. Psychotic symptoms were weakly associated with more severe depression at baseline in both cohorts. At follow-up, baseline psychotic symptoms were only associated with depressive symptoms in one sample; in the other, the effect size was close to zero. This supports the DSM5 system of psychotic symptoms being a separate code to severity rather than the ICD10 system which only allows the diagnosis of psychotic depression with severe depression. There was no clear support for psychotic symptoms being a baseline marker of treatment response.

## Introduction

Major depressive disorder is an important problem in adults and adolescents, with cumulative incidence in adolescence of 13% [1]. Adults with major depressive disorder (MDD) and psychotic features (delusions and/or hallucinations) have more severe symptoms [2, 3], a worse prognosis [4, 5], greater risk of relapse [5, 6] and a higher mortality [7]. More broadly, a recent large study in the UK demonstrated that psychotic experiences were associated with more severe depressive and anxiety symptoms and lower probability of recovery [8]. The role of psychotic symptoms in adolescent depression is less well studied. In the general population, there is a mean prevalence rate of 5% of subclinical psychotic symptoms [9]; compared to adults, these subclinical symptoms appear to be more common in children and adolescents, at around 7.5%, and are often transitory [10, 11]. Given these differences in prevalence of psychotic symptoms, it is possible that psychotic symptoms play a different role in adolescent compared with adult depression.

The two major international psychiatric classification systems view psychotic depression differently. ICD-10 assumes a unidimensional model, in which psychotic major depressive disorder can only be categorized under the ‘severe’ category [12]. This implies that psychotic symptoms cannot be found in mild/moderate depression. Contrastingly, the DSM5 has recently revised its classification: psychotic features can be coded alongside depression, but this is now separated from severity ratings [13]. It is unclear which viewpoint on psychotic depression is appropriate for depressed adolescents. This is important for clinicians, some of whom may assume that the presence of psychotic symptoms automatically means the depression is ‘severe,’ which may or may not be correct.

Psychotic depression at 12 years of age is associated with poorer educational, occupational and social outcomes by 16–20 years; however, it is not known whether this represents the likely outcome of psychotic symptoms across the adolescent years [14]. A recent symptom-level analysis of two adolescent to young adult (< 26 years old) community cohorts in the UK demonstrated that depressive and psychotic symptoms lie on the same continuum, with psychotic symptoms more common at the severe end [15]. This suggests that psychotic symptom emergence is an expression of clinical severity rather than a distinct category of affective disorder. If so then, as noted with adults, psychotic symptoms would be associated with poor treatment response.

We undertook secondary analyses using two cohorts recruited to randomized controlled trials of treatment for adolescent depression to compare the clinical features of depressed adolescents with and without psychotic symptoms at baseline and post-therapy follow-up. In particular, we looked for degree of correlation between severity of depressive symptoms and psychotic symptoms at baseline and follow-up. We tested whether psychotic symptoms at baseline:

- Were associated with current severity of depressive symptoms.
- Were associated with poor response to treatment.

## Methods

### Participants

Participants were recruited from two randomized controlled trials of depressed adolescents conducted in National Health Service specialist Child and Adolescent Mental Health clinics in the UK. As both trials showed no significant differences between treatment groups, participants from all treatment groups were combined within each dataset.

#### Adolescent depression antidepressants and psychotherapy trial (ADAPT)

ADAPT recruited 208 adolescents from the Cambridgeshire and Greater Manchester areas from 2000 to 2004 [16]. Inclusion criteria were: ages 11–17; current full or probable (at least four depressive symptoms) DSM-IV major depressive disorder; and significant social impairment. Individuals were excluded if they were not suitable to be in the treatment study: immediate admission required, significant learning disability, organic cause for depression, bipolar disorder, schizophrenia or selective serotonin reuptake inhibitors (SSRIs) contraindicated. Of note as per trial protocol in ADAPT (but not IMPACT), 34/249 (14%) of young people initially suitable for the study were excluded because they improved following a brief initial intervention.

Participants were randomized to selective serotonin reuptake inhibitor (SSRIs) plus routine psychosocial care (*n* = 103) or selective serotonin reuptake inhibitor (SSRIs), routine psychosocial care plus cognitive–behavioural therapy (CBT, *n* = 105). The routine psychosocial care delivered in both arms reflected NHS practice, including listening, support and problem solving. Treatment in both arms occurred throughout the 28-week study period, gradually reducing in frequency. Mean (sd) number of sessions in the two treatment arms were, respectively, 6.5 (4.0) and 10.6 (5.7). Through the study period (28 weeks), there were no statistically significant differences between the treatment groups on any clinical outcome (all *p* > 0.3) [17]

The study was approved and monitored by the North West Multi-Centre Research Ethics Committee and all local research ethics committees. Each participant and one adult with parental responsibility provided written informed consent.

#### Improving mood with psychoanalytic and cognitive therapies (IMPACT)

IMPACT recruited 465 adolescents from the Cambridgeshire, North London and Greater Manchester areas from 2010 to 2013 [18]. Adolescents aged 11–17 years with a DSM-IV diagnosis of unipolar major depressive disorder were recruited. Exclusion criteria included a primary diagnosis of either bipolar disorder, schizophrenia or an eating disorder, significant learning disability or pervasive developmental disorder, pregnancy, substance abuse disorders, selective serotonin reuptake inhibitor (SSRI) use contraindicated or previous completion of one of the study treatments.

Participants were randomized to cognitive behaviour therapy (CBT, *n* = 154), short-term psychoanalytic psychotherapy (STPP, *n* = 156) or a brief psychosocial intervention (BPI *n* = 155; this was a manualized form of the structured clinical care delivered to all participants in ADAPT). Median (IQR) number of sessions in each arm was less than planned: CBT 9 (5–14) over a mean (sd) of 25 (18) weeks; STPP 11 (5–23) over a mean (sd) of 28 (17) weeks; BPI 6 (4–11) over a mean (sd) of 28 (22) weeks. Participants in any arm were also permitted to receive SSRIs as part of their treatment in adherence with UK NICE guidelines for the treatment of unipolar depression (35%).

Participants were assessed at baseline, 6, 12, 36, 52 and 86 weeks post-randomization by assessors masked to treatment allocation. To enable comparison with the ADAPT study, this analysis will use data from baseline and 36 weeks (end of treatment period). At 36 weeks, self-reported depression symptoms did not differ significantly between any of the three treatment groups (all *p* > 0.06) [18].

The study was approved by the Cambridgeshire 2 Research Ethics Committee (reference 09/H0308/137) and local NHS provider trusts. All patients and their parents gave informed written consent.

### Instruments

#### Diagnosis: kiddie schedule for affective disorders and schizophrenia, present and lifetime version (K-SADS-PL)

The K-SADS-PL was used to measure whether DSM-IV diagnoses (major depression plus psychiatric comorbid disorders) were present, with scores brought together to a best estimate by consensus rating. Both participant and a parent were interviewed separately [19]. The psychotic symptoms group was defined as having delusions and/or hallucinations currently present at threshold level on the K-SADS-PL. Importantly, this measure was not able to distinguish whether psychotic symptoms were mood congruent/part of the depression itself. If data were missing on both items, or missing on one item and symptom absent on the other, the participant was excluded from analysis.

#### Depressive symptoms

The Mood and Feelings Questionnaire (MFQ) was used to measure participants’ self-reported depressive symptoms in both trials. Participants rated a set of 33 items (covering the range of DSM-IV depressive symptoms) over the last 2-week period [20]. The MFQ has good test–retest reliability, internal consistency and discriminant validity in clinical adolescent samples [21, 22].

#### Sensitivity analyses

Due to potential issues arising from self-rated questionnaires, analyses were repeated using the well-validated Children’s Depression Rating Scale—Revised, an observer-rated measure of depressive symptoms (only available for ADAPT) [23]. To test associations between psychotic symptoms and social function, analyses were repeated the sum of the social function items (HoNOSCA-Fx) of the Health of the Nations Outcome Scales for Children and Adolescents [HoNOSCA, items 5 (scholastic/language skills), 10 (peer relationships), 11 (self-care and independence), 12 (family), 13 (school attendance)]. The HoNOSCA is an observer-rated measure of mental health status, with 13 items across a range of symptom areas, behaviours and social function [24].

### Statistical analysis

Data analysis was performed using Stata version 14 (StataCorp, College Station, Tex.).

Only participants will full major depressive disorder and complete data for the psychosis variable at baseline were included. Depressive symptoms/functional baseline and follow-up were compared between those with and without baseline psychotic symptoms. Results were controlled for appropriate covariates, using structural equation modelling (ADAPT: region, age, gender; plus baseline value and treatment group at follow-up. IMPACT: treatment group, region, age, gender, ethnicity and SSRI treatment; plus baseline value, treatment group and time taken to start treatment at follow-up). To control for attrition, the full information maximum likelihood (FIML) option was performed.

## Results

192/208 of participants from ADAPT had full major depressive disorder at baseline. 190/192 (99%) from ADAPT and 439/465 (94%) from IMPACT had data on baseline psychotic symptoms. In ADAPT, 18 (9.5%) had threshold psychotic symptoms: 14 had hallucinations alone, and 4 had hallucinations plus delusions. In IMPACT, 42 (9.6%) had threshold psychotic symptoms: 28 had hallucinations alone, 8 had delusions alone, and 6 had hallucinations plus delusions. Table 1 shows baseline characteristics of both studies. Gender balance and prevalence of psychotic symptoms were similar in both studies. Ethnic diversity, baseline age and depression severity (as measured by MFQ score) were significantly higher in IMPACT compared to ADAPT. Proportion of participants with comorbid psychiatric disorders and impairment of social function was significantly higher for ADAPT.

**Table 1:** Baseline demographic and clinical variables for participants in the ADAPT and IMPACT studies

	ADAPT	IMPACT	Difference
Sample size	*n* = 190	*n* = 439	
Gender			
Male	47 (25%)	110 (25%)	*Χ*^2^(*df*1) = 0.01, *p* = 0.9
Female	143 (75%)	329 (75%)	
Age, mean (sd)	14.7 (1.2)	15.6 (1.4)	*Z* = 7.2, *p* < 0.00005
Baseline MFQ, mean (sd)	39.3 (11.6)	45.4 (10.5)	*Z* = 5.8, *p* < 0.00005
Baseline HoNOSCA-Fx, mean (sd)	10.6 (3.5)	6.7 (3.5)	*Z* = 11.0, *p* < 0.00005
Ethnicity* white	173/179 (97%)	345/431 (80%)	*Χ*^2^(*df*1) = 27, *p *< 0.0005
Number with at least one comorbid disorder	169 (89%)	213/439 (49%)	*Χ*^2^(*df*1) = 91, *p* < 0.0005
Number with psychotic symptoms	18 (9.5%)	42 (9.6%)	*Χ*^2^(*df*1) = 0.0, *p* = 1.0

*MFQ* Mood and Feelings Questionnaire. *HoNOSCA-Fx* Social function items (5, 10, 11, 12, 13) of the Health of the Nation Outcome Scales for Children and Adolescents

**Fig. 1:**
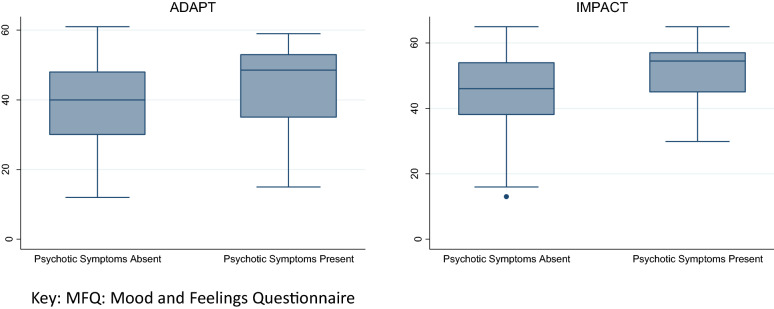
Baseline depressive symptoms in adolescents with and without psychotic symptoms

Univariate analysis (Table 2 and Fig. 1) demonstrated baseline self-rated depressive symptoms to be significantly higher in participants with psychotic symptoms in both studies (all *p* < 0.05). When controlling for relevant covariates (Table 2), this difference continued to be statistically significant in IMPACT (*β* = 0.143, *p* = 0.002) but not in ADAPT (*β* = 0.122, *p* = 0.087).

**Table 2:** Associations between baseline psychotic symptoms and baseline and post-treatment depressive symptoms/social function

Clinical measures	*N* for univariate	No. psychotic symptoms mean (sd)	Psychotic dymptoms present mean (sd)	Univariate comparison (*t* or Mann–Whitney *Z*)	Univariate *p*	Multivariate beta	Multivariate *p* (FIML)
ADAPT							
MFQ							
Baseline	190	38.8 (11.4)	44.1 (12.8)	*Z* = − 2.08	**0.04**	0.122	0.087
28 weeks	173	16.3 (14.7)	28.3 (19.2)	*Z* = − 2.45	**0.01**	0.171	**0.010**
CDRS-R							
Baseline	190	59.2 (10.1)	64.4 (7.4)	*Z* = − 2.21	**0.03**	0.174	**0.013**
28 weeks	175	34.5 (14.1)	44.3 (17.4)	*Z* = − 2.35	**0.02**	0.142	**0.028**
HoNOSCA-Fxn							
Baseline	190	10.4 (3.7)	12.2 (3.3)	*t* (*df* 188) = 2.05	**0.042**	0.157	**0.027**
28 weeks	176	6.9 (5.3)	8.0 (4.9)	*t* (*df* 174) = 1.1	0.3	0.055	0.4
IMPACT							
MFQ							
Baseline	439	45.2 (10.5)	51.3 (9.7)	*Z* = − 3.72	**0.0002**	0.143	**0.002**
42 weeks	299	26.5 (15.7)	29.8 (18.1)	*Z* = − 0.82	0.4	− 0.003	0.96
HoNOSCA-Fxn							
Baseline	396	6.8 (3.5)	6.4 (4.0)	*Z* = 0.5	0.6	− 0.035	0.5
42 weeks	226	3.4 (3.3)	3.5 (3.3)	*Z* = 0.2	0.8	− 0.006	0.9

Bold value indicates *p* < 0.05

*MFQ* Mood and Feelings Questionnaire, *CDRS-R* Children’s Depression Rating Scale—Revised, *HoNOSCA-Fx* Social function items (5, 10, 11, 12, 13) of the Health of the Nation Outcome Scales for Children and Adolescents

Time from baseline to post-treatment assessments [median (IQR)] for ADAPT was 28.3 (1.4) weeks and for IMPACT was 41.7 (4.1) weeks. In ADAPT and IMPACT respectively, post-treatment MFQ was complete for 173/190 (91%) and 299/439 (68%) participants included in the analysis. Univariate analysis demonstrated that participants with baseline psychotic symptoms had greater severity of depression post-treatment in ADAPT (*Z* = 2.45; *p* = 0.01) but not in IMPACT (*Z* = 0.8; *p* = 0.4, Table 2). After controlling for relevant covariates in FIML analysis (including baseline severity of depression), baseline psychotic symptoms were associated with higher post-treatment depressive symptoms in ADAPT (*β* = 0.171, *p* = 0.010, Table 2) but not in IMPACT (*β* = − 0.003, *p* = 0.96, Table 2). This analysis was repeated for IMPACT at the previous assessment point (median (IQR) 17.1 (4.1) weeks). Baseline psychotic symptoms continued not to be associated with follow-up depressive symptoms (*β* = − 0.004, *p* = 0.9).

Complete case analysis at follow-up revealed similar multivariate results: Baseline psychotic symptoms were associated with higher depressive symptoms in ADAPT (*n* = 173, *β* = 0.178, *p* = 0.010) but not IMPACT (*n* = 291, *β* = 0.007, *p* = 0.91). Results were similar for observer-rated depressive symptoms (CDRS-R) as self-rated symptoms (ADAPT only; baseline: *β* = 0.174, *p* = 0.013; follow-up: *β* = 0.142, *p* = 0.028). However for function (HoNOSCA-Fx), psychotic symptoms were associated with worse function at baseline in ADAPT (*β* = 0.157, *p* = 0.027) but not in IMPACT (*β* = − 0.035, *p* = 0.5); in both samples, baseline psychotic symptoms were not significantly associated with follow-up function (*p* > 0.4) (Table 2).

## Discussion

In this study, we wanted to explore the hypotheses that, in depressed adolescents without a primary diagnosis of bipolar disorder or schizophrenia, psychotic symptoms at baseline are: (a) strongly associated with severity of disorder and (b) a marker for poor treatment response. We used data from two independent cohorts, to allow replication.

In both cohorts, baseline depressive symptoms were higher in adolescents with psychotic symptoms. This remained significant when controlling for important confounders in the larger IMPACT sample, but was of borderline significance in ADAPT. Similar beta coefficients suggest that this may have been a type II error in ADAPT, especially as sensitivity analyses using the CDRS-R were significant. At first sight, this may seem to support the ICD-10 viewpoint that psychotic symptoms are only a feature of severe depression. However, and as can be seen clearly in Fig. 1, there was still great overlap between those with and without psychotic symptoms, and an adjusted standardized beta around 0.14 makes the effect size smaller than the standard 0.2 threshold for ‘small’ effects. The presence of significant numbers of young people with psychotic symptoms but depression at the milder end, and significant numbers without psychotic symptoms but severe depression supports the more flexible DSM5 viewpoint that psychotic symptoms can be coded at all levels of depression severity. Therefore, clinicians should not assume that psychotic symptoms mean that depression must be severe—instead they should measure severity directly. These findings are in keeping with recent evidence that psychotic experiences are seen across multiple mental disorders across the full spectrum of severity [25].

Findings on the prognostic implications of psychotic symptoms were mixed. In the ADAPT sample, psychotic symptoms were indeed associated with greater severity of depressive symptoms post-treatment, even when controlling for confounders including baseline severity. However, this finding was not replicated in IMPACT, and the very low standardized beta coefficient (of the opposite sign to expected: − 0.003) makes this very unlikely to be a type II error.

There are several possible explanations for the significant findings in one sample but not the other. The first is different sample characteristics, with there being a possibility of different predictive effects of psychotic symptoms within different ‘subtypes’ of depression. There were some significant differences between samples, although these operated in opposite directions, with greater severity of depressive symptoms in IMPACT, but higher levels of comorbidity and reduced social function in ADAPT. Also ADAPT had a higher proportion of white British participants and excluded the 14% of participants who responded to a brief intervention. And while all participants in ADAPT received SSRI antidepressants during the study, only about 30% of the IMPACT sample did. However, all participants received a psychological intervention in both studies: CBT, STPP or a Brief Psychosocial Intervention. Importantly, modality of psychological therapy had no effect on outcome. Importantly, prevalence of psychotic symptoms was the same in both samples (around 10%), and the zero effect size of psychotic symptoms on outcome in IMPACT makes it unlikely that a subgroup matching ADAPT would have a significant positive result. Second, the samples were recruited around ten years apart. Services may have had different referral thresholds. Differences in population behaviour may have led to differences, for example smoking of greater and more potent cannabis by British adolescents in recent years [26], which may make lead to a different ‘type’ of psychotic symptoms in depression. Although if this were the case in this sample, this would be likely to make psychosis rates different and, again, would be unlikely to account for an effect size of virtually zero. Third, post-treatment assessments occurred at different times after baseline (28 vs 42 weeks), and regression to the mean may have meant depressive symptoms no longer differed in our groups in the final IMPACT assessments; we therefore also compared groups at 17 weeks and again found no effect of baseline psychotic symptoms, making this explanation unlikely. The most likely explanation is that the finding in ADAPT was a chance observation, given non-replication in an independent (and much larger) sample, and lack of significant effects of baseline psychotic symptoms on social function at follow-up. Indeed, much has been written about the problem of ‘significant’ findings being published when they are in fact false; and replication is well recognized as being crucial to give us confidence that findings are in fact real [27]. This conclusion that results from the smaller ADAPT study could be a false positive is further supported by results from a recent meta-analysis of depressed adults, which found that the difference between post-follow-up depression severity in those with and without psychotic features was nonsignificant in studies with larger sample sizes (*n* cases > 50) [5]. It is also possible that differences in prognostic effects between adults and adolescents may be because of differences in the nature, aetiology and treatment responsivity of adolescent depression (in particular the fact that adolescent depression is usually first episode) [28], and differences in the nature of psychotic symptoms in adults and adolescents [10].

The contrasting results do not make it clear how depressed adolescents with psychotic symptoms should be treated—in IMPACT, the same treatments are as effective in reducing depressive symptoms in depression with and without psychotic features. However, this was not the case in ADAPT. There were however large reductions in depressive symptoms in both groups in both studies, so it is reasonable to use the same treatments as used in these RCTs to treat adolescents. Further larger studies of adolescents with depression with psychotic symptoms are needed to determine whether there is a need for additional treatment for such patients.

### Limitations

Both the ADAPT and IMPACT studies were initially designed as treatment comparison studies, and there were no primary hypotheses about the effects of psychotic symptoms. There is therefore a risk of type I errors in this secondary analysis. Crucially, psychotic symptoms were only asked about at as a current yes/no single question as part of the K-SADS-PL. This does not give any detail about the nature of the psychotic symptoms nor the time course (in particular whether the psychotic symptoms predated the depression). An important limitation of this is that our study could not determine whether these psychotic symptoms were specifically part of psychotic depression. While adolescents with bipolar disorder and schizophrenia were excluded from the study, it is possible that some adolescents had psychotic symptoms that predated the depression and may or may not have been part of a mental disorder. Our study can therefore only answer the question (itself useful) or what the implications of non-specific psychotic symptoms are on depressive symptoms. Furthermore, a continuous measure of psychotic symptoms would likely have had greater power than a simple yes/no question.

In IMPACT, a self-rated questionnaire of depressive symptoms only was used; however observer-rated and self-rated questionnaires led to similar results in ADAPT, and so this is unlikely to account for inaccuracies/differences in results. In addition, other potentially interesting explanatory variables were not enquired about, including childhood maltreatment, which is associated with psychotic symptoms, more severe depressive symptoms and poor prognosis [29]. While bipolar disorder was an exclusion criterion for both studies, some participants may have been in the early stages of bipolar disorder, with no manic episodes yet having occurred; again bipolar disorder is associated with psychotic symptoms, greater severity and poor prognosis; and bipolar disorder with psychosis is associated with a particularly poor prognosis [30, 31]. We also did not have information on lifetime course of depression, in particular presence of prior episodes, and effects may have differed in recurrent vs first episode depression. Neither study used a valid comprehensive measure of social function; therefore, we needed to use a non-validated short subscale; a more comprehensive validated measure may have provided significant results.

The psychotic symptom group sample sizes are also small in both ADAPT and IMPACT (< 10% of the sample size in both cases); this reduces the statistical power and the robustness of the results from the statistical analysis. The two cohorts did have different findings; while it is more appropriate to make the conservative null conclusion we have done, this may be incorrect and result need replication in a third sample. In addition, participants in both studies, as with all RCTs, were not representative of the community population (in particular because they were seeking help and agreed to being in a treatment study)—therefore, results may not generalize to depressed adolescents in general.

To answer these questions authoritatively, a study needs to collect a larger proportion of adolescents with psychotic symptoms and measure psychotic symptoms using detailed and well-validated scales, with details on longitudinal pattern of depressive episodes and psychotic symptoms.

### Clinical implications

There is a large overlap in severity between depressed adolescents with and without psychotic symptoms, and clinicians should not assume that psychotic symptoms necessarily mean severe depression and worse outcome. It is reasonable to continue to use evidence-based psychological therapies with or without an SSRI to treat adolescents with depression.

## Data Availability

The data that support the findings of this study are available from the corresponding author PW, upon reasonable request.

## Code availability

The code is available from the corresponding author by request.
